# Photosynthetic Response of an Alpine Plant, *Rhododendron delavayi* Franch, to Water Stress and Recovery: The Role of Mesophyll Conductance

**DOI:** 10.3389/fpls.2015.01089

**Published:** 2015-12-08

**Authors:** Yanfei Cai, Jihua Wang, Shifeng Li, Lu Zhang, Lvchun Peng, Weijia Xie, Feihu Liu

**Affiliations:** ^1^School of Agriculture, Yunnan UniversityKunming, China; ^2^Flower Research Institute of Yunnan Academy of Agricultural SciencesKunming, China; ^3^National Engineering Research Center for Ornamental HorticultureKunming, China

**Keywords:** mesophyll conductance, photosynthetic limitation, recovery, *Rhododendron delavayi*, stomatal conductance, water stress

## Abstract

*Rhododendron delavayi* Franch is an evergreen shrub or small tree with large scarlet flowers that makes it highly attractive as an ornamental species. The species is native to southwest China and southeast Asia, especially the Himalayan region, showing good adaptability, and tolerance to drought. To understand the water stress coping mechanisms of *R. delavayi*, we analyzed the plant's photosynthetic performance during water stress and recovery. In particular, we looked at the regulation of stomatal (*g*_s_) and mesophyll conductance (*g*_m_), and maximum rate of carboxylation (V_cmax_). After 4 days of water stress treatment, the net CO_2_ assimilation rate (A_N_) declined slightly while *g*_s_ and *g*_m_ were not affected and stomatal limitation (S_L_) was therefore negligible. At this stage mesophyll conductance limitation (MC_L_) and biochemical limitation (B_L_) constituted the main limitation factors. After 8 days of water stress treatment, A_N_, *g*_s_, and *g*_m_ had decreased notably. At this stage S_L_ increased markedly and MC_L_ even more so, while B_L_ remained relatively constant. After re-watering, the recovery of A_N_, *g*_s_, and *g*_m_ was rapid, although remaining below the levels of the control plants, while V_cmax_ fully regained control levels after 3 days of re-watering. MC_L_ remained the main limitation factor irrespective of the degree of photosynthetic recovery. In conclusion, in our experiment MC_L_ was the main photosynthetic limitation factor of *R. delavayi* under water stress and during the recovery phase, with the regulation of *g*_m_ probably being the result of interactions between the environment and leaf anatomical features.

## Introduction

Low water availability is considered as one of the main environmental factors limiting plant growth and productivity worldwide (Chaves et al., 2009). The majority of climate change scenarios predict an increase in drought incidents throughout the world (Lemke et al., 2007). Thus, the strategies of tolerance, adaption, and survival will be of major importance for plants growing under water stress. It has been shown that water stress primarily affects photosynthetic CO_2_ assimilation, and therefore limits plant productivity and growth (Flexas et al., 2006). The response of photosynthesis to water stress has received considerable attention in recent decades, especially the key factors limiting photosynthesis under water stress conditions (Flexas et al., 2002; Lawlor and Cornic, 2002). However, there is an on-going debate about whether the determinant for photosynthesis under water stress conditions is stomatal closure, diffusive resistance, or metabolic uncoupling (Lawlor and Cornic, 2002; Flexas et al., 2009, 2014; Pinheiro and Chaves, 2011; Campos et al., 2014; Chen et al., 2015).

Stomatal closure is generally considered as the initial and main cause of the decrease in photosynthesis under water stress conditions, as diffusion of CO_2_ from the atmosphere to the sites of carboxylation in the chloroplast is impaired (Flexas et al., 2009; Rho et al., 2013). However, reduced leaf CO_2_ diffusion conductance is not only due to stomatal closure, but also due to the decreased internal conductance of CO_2_ diffusion (mesophyll conductance, *g*_m_; Galmés et al., 2007; Zhou et al., 2014; Chen et al., 2015). As a result of the recognition of the importance of *g*_m_, the studies pertaining to *g*_m_ have increased exponentially in recent years (Zhou et al., 2007; Flexas et al., 2009; Galle et al., 2009; Niinemets et al., 2009; Rancourt et al., 2015). Some studies suggested that *g*_m_ was consistently delayed by a few days compared with *g*_s_ (Rancourt et al., 2015), while other studies suggested that *g*_m_ can vary at least as fast as *g*_s_, and *g*_m_ contributes greatly to the limitation of photosynthesis during water stress and recovery after water stress (Gomes et al., 2008). Recently, the work by Carriquí et al. (2015) suggested that *g*_s_, and *g*_m_ were co-responsible for the lower photosynthesis observed in ferns as compared with angiosperms, and that *g*_m_ was the most constraining factor for photosynthesis in ferns. These findings support the idea of an important role for *g*_m_ in the photosynthetic responses of plants to climatic constraints.

Additionally, photosynthesis also decreases due to metabolic impairments and/or cell damage, especially under severe water stress combined with intensive light and high temperature (Flexas et al., 2006; Zhou et al., 2007). Under these circumstances, down-regulation of photosynthesis increases the production of reactive oxygen species (Takahashi and Murata, 2005) and even leads to photoinhibition (Massacci et al., 2008; Wang et al., 2012; Chastain et al., 2014). Furthermore, the carbon balance of a plant enduring a water stress period depends as much on the rate and degree of photosynthetic recovery (Flexas et al., 2006). The capability for photosynthetic recovery after exposure to water stress conditions is essential to understand the plant response to water stress, and to determine appropriate irrigation in agricultural practices. Many studies have addressed the response of photosynthesis to water stress, but the underlying process of photosynthetic recovery from water stress is poorly understood. In particular, knowledge about the factors limiting photosynthesis under these conditions, and their possible interactions with other environmental conditions are scarce (Flexas et al., 2009; Campos et al., 2014).

A previous study proposed a method which divides the total limitation into stomatal limitation (S_L_), mesophyll conductance limitation (MC_L_), and biochemical limitation (B_L_; Grassi and Magnani, 2005). Galmés et al. (2007) found that MC_L_ was the main limiting factor for photosynthesis recovery after re-watering in 10 Mediterranean species. The results of Flexas et al. (2009) suggested that stomatal closure recovered much more slowly than *g*_m_, thus photosynthesis recovery after re-watering was mostly limited by S_L_. These findings underline the importance of *g*_m_ during water stress conditions, and further suggest an important contribution to the overall adaptation of plants to drought stress conditions. However, current knowledge about the physiological limitations to photosynthesis during short-term water stress and recovery after re-watering is scarce (Flexas et al., 2009, 2014). It would be necessary to apply this method to analyze photosynthetic limitations in plants subjected to water stress, especially in circumstances pertaining to a shorter period of water stress and recovery after re-watering.

*Rhododendron* is one of the most well-known alpine flowers. Of the approximately 571 *Rhododendron* species in China, 320 species are found within the Yunnan Province of southwestern China (Fang et al., 2005). Most of the above *Rhododendron* species are distributed in alpine areas and commonly are less constrained by water shortage. However, the five consecutive years of spring drought experienced in Southwest China since 2009 have had great adverse effects on the growth and flowering of *Rhododendron*. Furthermore, droughts frequently occur in winter and spring, water supply limitation is gradually becoming one of the dominant limitations for the growth and application of *Rhododendron*. Currently, little is known about the physiological responses during the process of water stress and the recovery after re-watering.

*Rhododendron delavayi* Franch is an evergreen shrub or small tree with large scarlet flowers that makes it highly attractive as an ornamental species. The species is native to southwest China and southeast Asia, especially the Himalayan region. The leaf of *R. delavayi* is leathery, and the abaxial surface with 1-layered, spongy or somewhat agglutinated, whitish to fawn indumentums (Fang et al., 2005). In our previous study, we found that the stomata of *R. delavayi* exist only on the abaxial surface. When compared with *R. irroratum* and *R. yunnanense* which grow under the same conditions, *R. delavayi* exhibited perfect adaptability and tolerance to dry and high radiation environments, including traits such as with smaller stomata, larger stomatal density, and higher ratio of palisade and spongy tissue (Cai et al., 2014). The aim of the present work was to evaluate the responses of photosynthesis to water stress and recovery, and analyze the main limiting factors of photosynthesis during water stress and recovery, emphasizing the leaf internal diffusive components. Our hypotheses were that: (1) excitation pressure imposed by water stress will cause a general decline of the photosynthetic performance; (2) down-regulation of mesophyll conductance to CO_2_ may impose a similar or even greater limitation to photosynthesis than that imposed by stomatal closure during water stress treatment; (3) photosynthetic recovery after re-watering may be mostly limited by mesophyll conductance limitations.

## Materials and methods

### Plant materials and treatments

The experiment was carried out in a greenhouse in Kunming, China (a1t1926 m, E 102°46′, N 25°07′). Five-year-old plants of *R. delavayi* were grown in 9-L plastic pots (one plant per pot) filled with a mixture of red soil and humus (V/V, 1/3). The plants were housed in the experimental greenhouse under natural light and temperature conditions. From budbreak (20 March) to the beginning of the experimental period (25 June), the plants were irrigated three times per week to maintain sufficient water supply. Then, 30 plants were placed in the same greenhouse, and subdivided randomly into two groups: the control and the stressed plants. The control plants were irrigated daily to field capacity, while the treatment plants were not irrigated. Ten days after stopping the irrigation, the leaf stomatal conductance (*g*_s_) decreased to 0.02 mol H_2_O m^−2^s^−1^ (severe water stress), at which point all of the plants were re-watered to field capacity for recovery.

### Relative water content (RWC)

Plant water status was assessed by relative water content (RWC) on the first whorl of the leaves. In order to determine RWC, four fresh leaves per replication were collected and their fresh weights (FW) were obtained. Next, these leaves were placed in water to float for 24 h at 4°C in the dark to obtain their turgid weights (TW). The leaves were then oven dried at 72°C for 48 h to obtain their dry weights (DW). RWC was determined by the formula: RWC(%) = (FW − DW)/(TW − DW) × 100 (de Souza et al., 2013).

### Soil moisture content (SMC)

In order to determine soil moisture content (SMC), the soil of four pots per replication were collected in an aluminum box and their fresh weights (FW) determined. The soil sample was then oven dried at 105°C for 48 h to obtain their dry weights (DW). SMC was determined by the formula: SMC(%) = (FW−DW)/DW×100.

### Instantaneous gas exchange

Instantaneous gas exchange measurements were tested daily, between 12:00 and 13:00 h local time, using an open gas-exchange system (Li-6400XT; Li-Cor, Inc., Nebraska, USA) equipped with a light source (Li-6400-02B). No measurements were taken on day 6 due to technical problems with the Li-6400. All measurements were made on the young, fully expanded leaves at a saturating photosynthetic photo flux density (PPFD) of 1000 μmol m^−2^s^−1^, with a CO_2_ concentration of 400 μmol mol^−1^ in the leaf cuvette. During the instantaneous measurements, net CO_2_ assimilation rate (A_N_), stomatal conductance (*g*_s_), intercellular CO_2_ concentration (C_i_), transpiration rate (T_r_), air temperature (T_*air*_), and leaf-to-air vapor pressure deficit (VPD) were recorded automatically by the Li-6400XT.

### CO_2_ response curve and chlorophyll fluorescence

The CO_2_ response curve (A_N_–C_i_ curves) and chlorophyll fluorescence were measured simultaneously with a combined open gas exchange system and chlorophyll fluorescence system (Li-6400-40; Li-Cor Inc., Nebraska, USA) on four specific sampling days for each treatment: day 4 and day 8 after stopping the irrigation, and then day 1 and day 3 after re-watering.

A_N_–C_i_ curves were constructed as a function of the ambient CO_2_ concentration (C_a_) ranging from 50 to 2000 μmol mol^−1^. Light intensity was maintained at 1000 μmol m^−2^ s^−1^. The flow rate within the chamber was controlled at 500 mmol air min^−1^, and the block temperature at 25°C. VPD was kept within a variation of 0.5 kPa during the performance of a single curve. At first, C_a_ in the leaf chamber decreased stepwise from 400 to 50 μmol mol^−1^. After that C_a_ was returned to 400 μmol mol^−1^ to restore the original A_N_. Then, C_a_ was increased stepwise until 2000 μmol mol^−1^ to complete the curve. The number of different C_a_ values used for the response curve was 13, and the time lag between two consecutive measurements at each C_a_ was restricted to 2–4 min. The A_N_-C_i_ curves were used to estimate the maximum carboxylation rate of Rubisco (V_cmax_), the maximum electron transport rate (J_max_) used a utility developed by Sharkey et al. (2007), and was based on an alternative A_N_-C_i_ curve fitting method through a non-rectangular hyperbola version of the model provided by Farquhar et al. (1980).

For the chlorophyll fluorescence measurements, the leaf was irradiated by an actinic radiation of 1000 μmol m^−2^ s^−1^ (90–10% red-blue light) for 15–20 min until stable photosynthesis occurred. Then the steady state fluorescence (F_s_) was recorded and subsequently another saturating light pulse around 6000 μmol m^−2^s^−1^ was applied to determine the maximum fluorescence (F${}_{\text{m}}^{\prime }$). Actinic light was removed and the leaves were irradiated with far-red light to obtain F_o_ adapted to light (F${}_{\text{o}}^{\prime }$). From these values, the photochemical quenching (qP) was calculated as: $\text{qP}=\left(F_{m}^{\prime }-F_{s}\right)/\left(F_{m}^{\prime }-F_{\text{o}}^{\prime }\right)$, the actual photochemical efficiency of photosystem II was calculated as: ($(\Phi \text{PSII})=(F_{m}^{\prime }-F_{s})/F_{m}^{\prime }$; Genty et al., 1989), and the electron transport rate was calculated as: (J_flu_) = ΦPSII × PPFD × 0.5 × 0.85 (Valentini et al., 1995).

After an adaptation period of 30 min in the dark, the minimum fluorescence (F_o_) was measured with the light which was sufficiently low to avoid a photochemical reaction. The maximum fluorescence (F_m_) was obtained by applying a saturating light pulse of 6000 μmol m^−2^ s^−1^ for 0.8 s. The maximum quantum efficiency of photosystem II (F_v_/F_m_) was calculated as: F_V_/F_m_ = (F_m_−F_o_)/F_m_(Genty et al., 1989).

### Determination of mesophyll conductance

Mesophyll conductance (*g*_m_) was calculated using the “variable” method as described by Harley et al. (1992):

$$\begin{array}{l} g_{m}=\frac{A_{N}}{C_{i}-\frac{\Gamma^{*}\left(J_{flu}+8\left(A_{N}+R_{d}\right)\right)}{J_{flu}-4\left(A_{N}+R_{d}\right)}} \end{array}$$

where, A_N_ and C_i_ are taken from A_N_–C_i_ curve, and J_flu_ was estimated from chlorophyll fluorescence on the same leaf, and Γ* was 37.43 μmol mol^−1^ at 25°C according to Bernacchi et al. (2002). R_d_ was respiration in the light and determined according to the method of Laisk (1977). *g*_m_ values were calculated for measurements of the net assimilation rate at a C_i_ level of 100–300 μmol mol^−1^, and the average value of *g*_m_ was determined for each leaf. The calculated values for *g*_m_ were used to estimate the chloroplast CO_2_ concentration (C_c_) as: C_c_ = C_i_−(A_N_/*g*_m_)

### Photosynthetic limitation analysis

To compare photosynthetic limitations during water stress and recovery, the approach proposed by Grassi and Magnani (2005) was used to partition photosynthetic limitation into three components related to stomatal conductance (S_L_), mesophyll conductance (MC_L_), and leaf biochemical characteristics (B_L_). This requires assuming a reference which had a maximum assimilation rate. In the current study, the maximum assimilation rate, concomitant with *g*_s_, *g*_m_, and V_cmax_, was generally found under well-watered conditions. Therefore, the control treatment was used as a reference. According to this method, non-stomatal limitations were defined as the sum of the contributions of mesophyll conductance and leaf biochemistry (NS_L_ = MC_L_ + B_L_), while the diffusive limitations were the sum of the contributions of the stomatal and mesophyll conductance (D_L_ = S_L_+MC_L_).

## Results

### Experimental conditions and plant water stress

**Figure 1:**
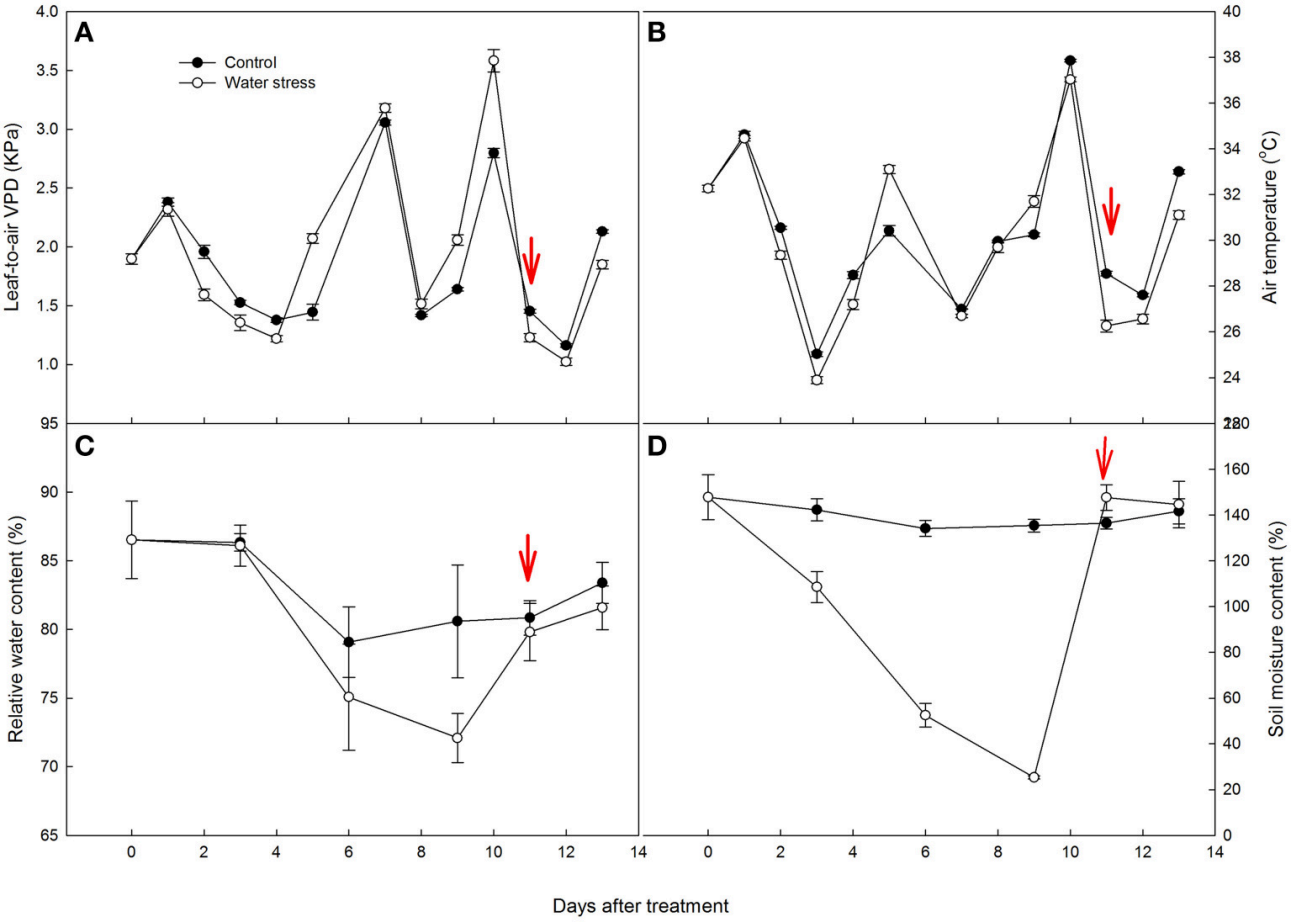
**Variations of (A) leaf-to-air vapor pressure deficit (VPD), (B) air temperature (T_***air***_), (C) leaf relative water content (RWC), and (D) soil moisture content (SMC) during the experiment**. The values represent means ± SE (*n* = 10 for **A,B**, *n* = 4 for **C,D**). Day 0 corresponds to the first day of water stress and the arrow indicates the start of re-watering.

During the experiment VPD of the control and stressed plants remained mostly above 1.5 kPa. Air temperature (T_*air*_) was between 23.9 and 37.9°C (Figures 1A,B). The highest VPD and T_*air*_ corresponded to the most severely stressed plants without irrigation for 10 days. For the three days (3, 7, and 11 days after the onset of the experiment) with clouds or rainfall, T_*air*_ values of all of the plants were close to 25°C, VPD also decreased regardless of the treatments.

During the experiment period, RWC in the control plants had some fluctuation, with an average of 82.8%. After 6 days of water stress, RWC in the stressed plants decreased slightly. After 9 days of water stress, RWC in the stressed plants decreased to 72.1%, and there was a significant difference between the control and stressed plants. After re-watering, RWC in the water stressed plants increased to values similar to those in the control plants (Figure 1C).

During the experiment period, SMC of the control plants was between 134.1 and 147.8%, while SMC of the stressed plants decreased significantly, from 147.8% at the beginning of the treatment to 25.3% after 9 days of water stress (Figure 1D).

### Photosynthetic responses to water stress and recovery

**Figure 2:**
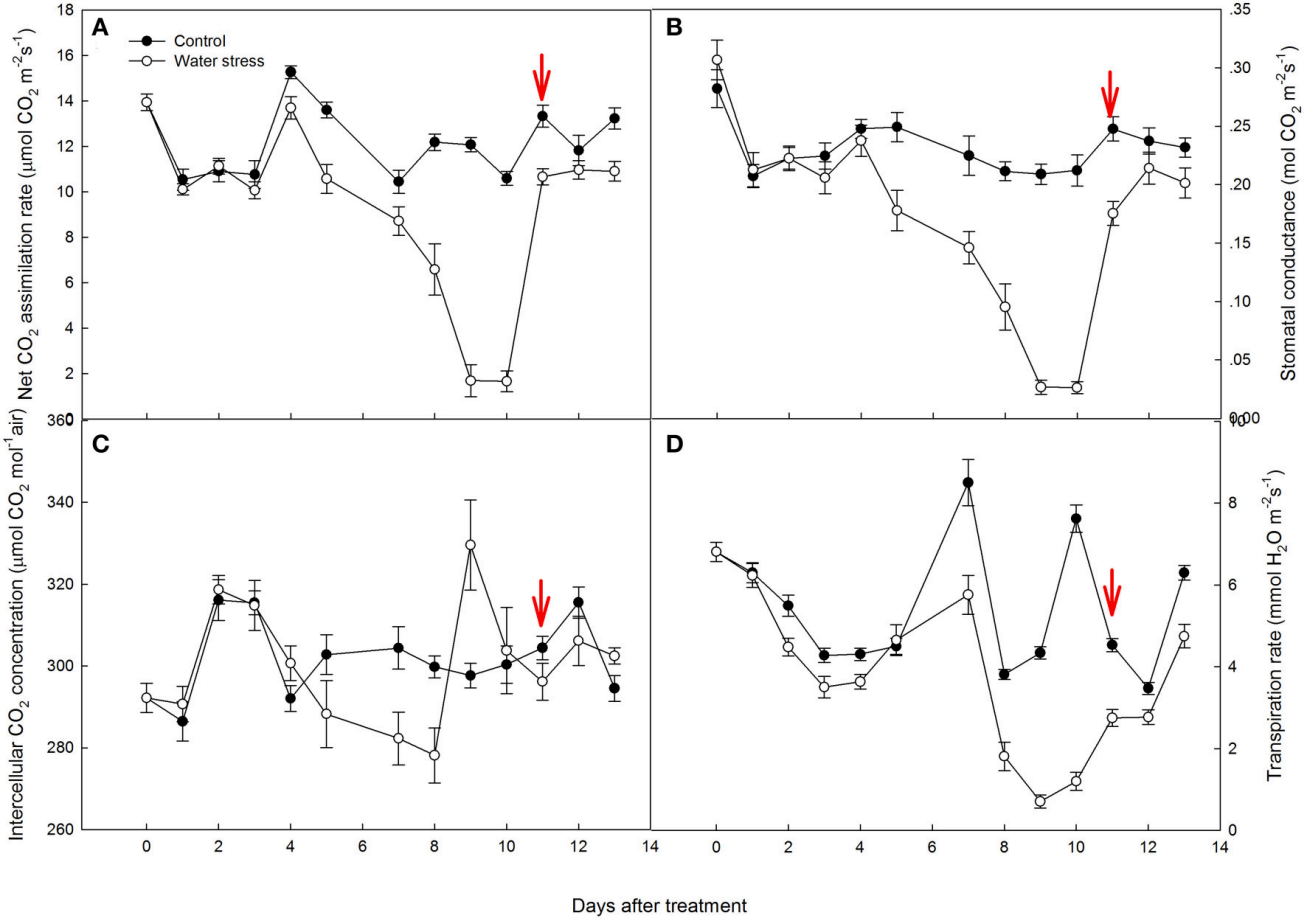
**Variations of (A) net CO_2_ assimilation rate (A_N_), (B) stomatal conductance (***g***_s_), (C) intercellular CO_2_ concentration (C_i_), and (D) transpiration rate (T_r_) during the experiment**. Values represent means ± SE (*n* =10). Day 0 corresponds to the first day of water stress and the arrow indicates the start of re-watering.

A_N_ of the control plants oscillated during the experiment, from 15.2 μmol CO_2_ m^−2^s^−1^ by day 4 to 10.5 μmol CO_2_ m^−2^s^−1^ by day 7. Water stress caused a slight reduction in A_N_ from day 1 to day 7, and A_N_ of the stressed plants was more than 80% of the control plants. However, water stress resulted in a significant reduction in A_N_ by day 8, and the value of A_N_ rapidly decreased to 1.68 μmol CO_2_ m^−2^s^−1^ by day 9, only 14% of the value of the control plants. After 1 d of re-watering, A_N_ was restored to 80% of the control value, but no further recovery of A_N_ was observed afterwards (Figure 2A).

Stomatal conductance (*g*_s_) showed a similar variation to A_N_. In the control plants, *g*_s_ were maintained above 0.20 mol CO_2_ m^−2^s^−1^, with an average value of 0.22 mol CO_2_ m^−2^s^−1^. The stressed plants had similar *g*_s_ values to the control plants from day 1 to day 4. Water stress caused a gradual decline in *g*_s_ from day 5 to day 8, and *g*_s_ values of the stressed plants were approximately 0.10 mol CO_2_ m^−2^s^−1^ by day 8. However, *g*_s_ quickly declined to 0.02 mol CO_2_ m^−2^s^−1^ by day 9. Like A_N_, the recovery of *g*_s_ after re-watering was very quick, restored to 70% of the control value in the first day, and restored to 86% of the control plants after 3 days of re-watering (Figure 2B).

As a consequence of the decreased *g*_s_ during water stress treatment, C_i_ and T_r_ were also depressed (Figures 2C,D). It is worth noting that the change in C_i_ did not simply follow that in *g*_s_. During the last 2 days of water stress treatment, C_i_ increased quickly and exceeded the control plants. After re-watering, C_i_ decreased and maintained lower values than that observed in the control plants.

When compared with the control plants, V_cmax_ and J_max_ in the stressed plants were reduced by 22 and 14% by day 4, respectively. However, V_cmax_ and J_max_ did not decrease further after 8 days of water stress treatment. After re-watering, V_cmax_ and J_max_ increased to the levels equivalent to that in the control plants, and even higher than the control plants after 3 days of re-watering (Table 1).

**Table 1:** **Net CO_2_ assimilation rate (A_N_), stomatal conductance (***g***_s_), Rubisco maximum carboxylation rate (V_cmax_), maximum electron transport rate (J_max_), mesophyll conductance (***g***_m_) and chloroplast CO_2_ concentration (C_c_) under water stress and re-watering**.

	**A_N_ (μ mol CO_2_ m^−2^s^−1^)**	***g*_s_ (mol CO_2_ m^−2^s^−1^)**	**V_cmax_ (μ mol CO_2_ m^−2^s^−1^)**	**J_max_ (μ mol e^−1^ m^−2^s^−1^)**	***g*_m_ (mol CO_2_ m^−2^s^−1^)**	**C_c_ (μ mol CO_2_ mol^−1^ air)**
**TREATMENTS AFTER WITHOUT IRRIGATION**						
Control	15.26±0.28^a^	0.25±0.01^a^	33.71±1.18^a^	144.59±5.83^ab^	0.074±0.013^a^	121.43±8.78^a^
Stress (4)	13.69±0.49^b^	0.24±0.01^a^	26.34±1.11^bc^	124.92±5.29^c^	0.069±0.003^ab^	122.35±15.86^a^
Control	12.18±0.36^bc^	0.21±0.01^b^	32.62±1.52^a^	148.93±1.88^ab^	0.070±0.012^a^	120.35±10.63^a^
Stress (8)	6.59±1.13^d^	0.09±0.02^d^	24.74±0.95^c^	123.07±3.48^c^	0.037±0.012^b^	106.42±1.37^a^
**TREATMENTS AFTER RE-WATERING**						
Control	13.33±0.49^*b*^	0.25±0.01^a^	33.08±3.64^a^	153.75±5.51^a^	0.057±0.014^ab^	125.49±6.92^a^
Recovery (1)	10.66±0.36^c^	0.18±0.01^c^	27.23±0.84^bc^	118.87±2.14^c^	0.060±0.013^ab^	137.20±10.41^a^
Control	13.22±0.46^b^	0.23±0.01^ab^	30.21±1.09^abc^	128.51±5.72^c^	0.087±0.019^a^	122.09±12.21^a^
Recovery (3)	10.90±0.43^c^	0.20±0.01^bc^	30.40±2.64^ab^	135.67±10.06^bc^	0.054±0.004^ab^	103.03±7.52^a^

*Data are means ± SE. Numbers in brackets on the first part show days of water stress whereas in the second part indicate days of re-watering. Different letters in the same column indicate statistical difference (p < 0.05)*.

Mesophyll conductance (*g*_m_) did not decline by day 4, but suddenly decreased to 53% of the control plants by day 8. However, *g*_m_ totally recovered during the first day of re-watering. As a consequence of the decreased *g*_m_ during water stress treatment, C_c_ was also depressed. The depression was more remarkable by day 8. After re-watering, due to the rapid recovery of *g*_m_, C_c_ increased and was higher than the control plants during the first day after re-watering (Table 1).

### Change in chlorophyll fluorescence during water stress and recovery

**Figure 3:**
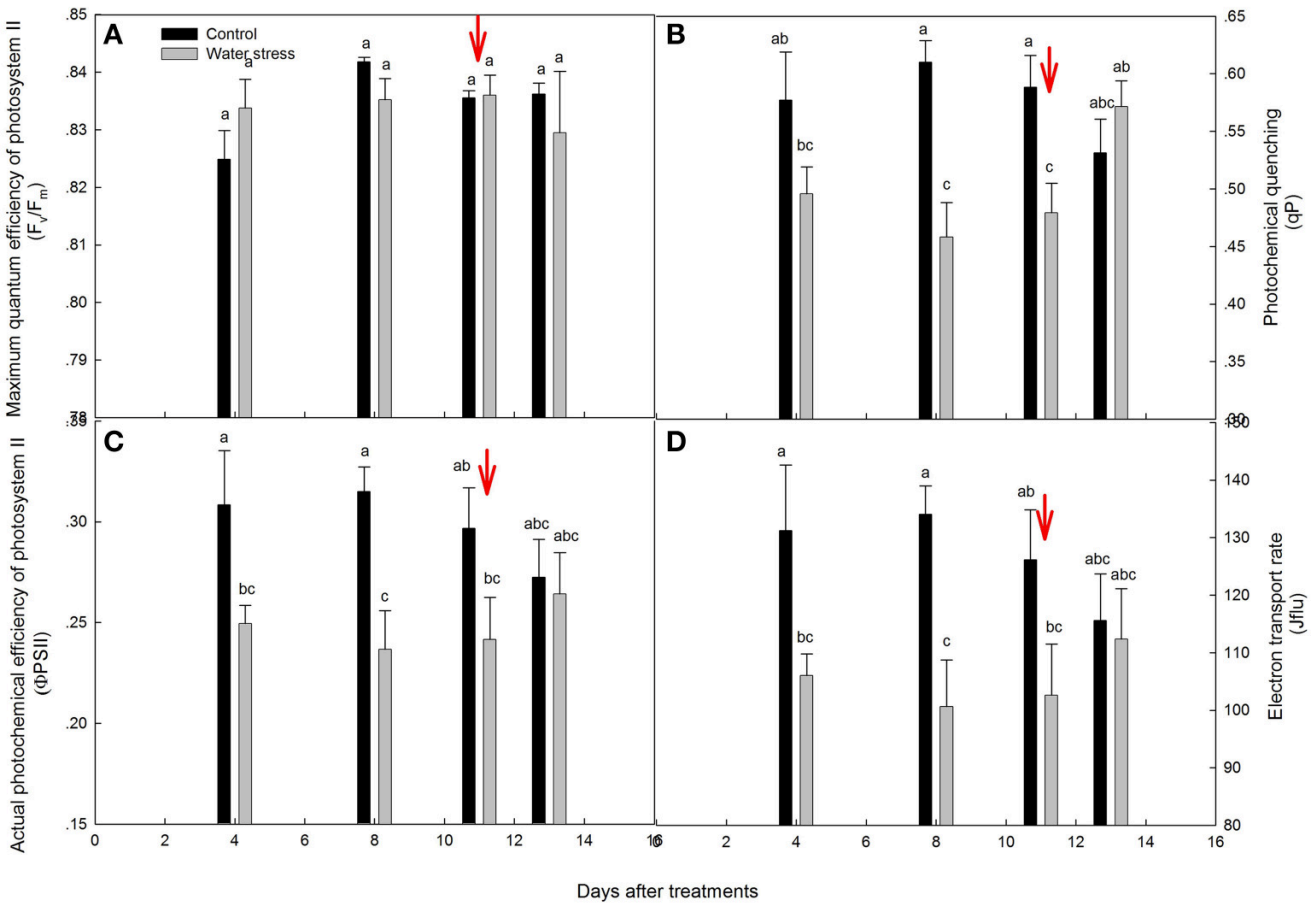
**Variations of (A) maximum quantum efficiency of photosystem II (F_v_/F_m_), (B) photochemical quenching (qP), (C) actual photochemical efficiency of photosystem II (ΦPSII), and (D) electron transport rate (J_flu_) during the experiment**. Values represent means ± SE (*n* = 4). Day 0 corresponds to the first day of water stress and the arrow indicates the start of re-watering.

The chlorophyll fluorescence parameters are shown in Figure 3. The values for F_v_/F_m_ were kept above 0.82 throughout the experimental period, and were not significantly different between the control plants and stressed plants (Figure 3A). By day 4, the values for ΦPSII, qP, and J_flu_ of the stressed plants significantly declined, and further declined by day 8. After re-watering, the recovery of ΦPSII, qP, and J_flu_ was slight on the first day, and these recovered progressively to the levels seen in the control plants within 3 days (Figures 3B–D).

### Photosynthetic limitations during water stress and recovery

After 4 days of water stress treatment, S_L_ was negligible, while MC_L_ and B_L_ imposed some limitations to photosynthesis. With the further imposition of water stress treatment, S_L_ and MC_L_ increased rapidly, while B_L_ did not increase by day 8. It is worth noting that MC_L_ was more than two times the level observed for S_L_. After 1 d of re-watering, S_L_ and MC_L_ decreased rapidly, while B_L_ did not change. After 3 days of re-watering, S_L_ and B_L_ further declined, while MC_L_ remained stable, and thus MC_L_ was the main limitation to photosynthesis during recovery (Table 2).

**Table 2:** **Photosynthetic limitation of photosynthesis under water stress and re-watering**.

**Treatments**	**Limitations (%)**					
	**Stomatal limitation (S_L_)**	**Mesophyll conductance limitation (MC_L_)**	**Biochemical limitation (B_L_)**	**Diffusive limitation (D_L_)**	**Non-stomatal limitation (NS_L_)**	**Total limitation (T_L_)**
Stress (4)	0.5	6.7	10.7	7.4	17.4	17.9
Stress (8)	11.9	27.9	8.0	39.8	35.9	47.8
Recovery (1)	4.0	11.1	8.8	15.1	19.9	23.9
Recovery (3)	2.5	16.9	3.6	19.4	20.5	23.0

*Numbers in brackets show days of water stress or re-watering*.

## Discussion

### Photosynthetic responses to water stress and recovery

The water stress-induced decline of A_N_, *g*_s_, and *g*_m_ was always slower than the recovery after re-watering. After 4 days of water stress treatment, A_N_ declined slightly, and there was almost no change in the values for *g*_s_ and *g*_m_. As the duration of the water stress treatment increased, A_N_, *g*_s_, and *g*_m_ decreased markedly by day 8. After 9 days of water stress, the values of A_N_ was close to zero, *g*_s_ reached 0.02 mol CO_2_ m^−2^s^−1^. There was no further reduction in the next continued 2 days drought. After re-watering for 1 day, no plant died and all of them showed some recovery. Results suggested that *g*_s_ can use as a reference parameter to justify the levels of water stress of *R. delavayi*, i.e., severe water stress (*g*_s_ near 0.02 mol CO_2_ m^−2^s^−1^). Flexas et al. (2009) also use *g*_s_ to define the levels of water stress of *Vitis* hybrid Richter-110, i.e., moderate water stress (*g*_s_ near 0.15 mol CO_2_ m^−2^s^−1^) and severe water stress (*g*_s_ near 0.05 mol CO_2_ m^−2^s^−1^).

Once water stress was established and maintained, *g*_s_ was more stable than *g*_m_. An intriguing behavior of *g*_m_ was a total recovery after irrigation for 1 day, while *g*_s_ and A_N_ were restored to above 70% of the control values. Throughout the periods of water stress and recovery, A_N_ and *g*_s_ of followed the same course, indicating a strong co-regulation of these parameters, as shown in many studies (Chaves et al., 2002; Lawlor and Cornic, 2002). However, with the further recovery of *g*_s_ and A_N_, the values for *g*_m_ were not restored further, and even slightly decreased after re-watering for 3 days, indicating an independent regulation for *g*_s_ and *g*_m_. Previous studies suggested that *g*_s_ appears to be more independent of environmental conditions except for VPD (Pou et al., 2008; Hanson et al., 2013), and the opening and closing of stomata is regulated by the integration of environmental signals and endogenous hormonal stimuli (Daszkowska-Golec and Szarejko, 2013). However, the response of *g*_m_ to water stress strongly depends on the water channels aquaporins (Daszkowska-Golec and Szarejko, 2013), and the impact of additional environmental factors, especially light condition (Zhou et al., 2007). Regulatory mechanisms such as the phosphorylation of aquaporins can be light dependent (Tournaire-Roux et al., 2003). The results of Galle et al. (2009) further showed that *g*_m_ declines considerably and recovers slightly under high light conditions; while under low light conditions it does not decrease under water stress. In the present study, the first day of re-watering was cloudy with rain, and T_*air*_ was close to 25°C with a decreasing VPD. Considered together with the present results, it seems that the adaptation of *g*_m_ to water stress and its rapid recovery after rewatering is related to additional factors, such as light intensity and temperature, and suggests the necessity to better understand the regulation of mesophyll conductance, which conceivably depends on the environmental conditions.

In plants under water stress treatment, stomatal closure results in rapid decrease of *g*_s_ and A_N_ (Campos et al., 2014). However, non-stomatal factors were also important for the regulation of photosynthetic capacity, as reflected by both the reduction of V_cmax_ and J_max_. The decrease in V_cmax_ is mostly due to the reduced activity of fructose-1,6-biphosphate phosphatase, as well as the decreased activity of Rubisco (Maroco et al., 2002; Bota et al., 2004). However, recent transcriptomic analysis in plants subjected to water stress showed that some genes related to Rubisco activase, Calvin cycle enzymes, and PSI and PSII, are conversely up-regulated during the acclimation to water stress (Cramer et al., 2007; Song et al., 2014). Proteomic analysis also confirmed that some photosynthetic proteins such as notably Rubisco and sedoheptulose 1,5-bisphosphatase, and mitochondrial glycine decarboxylase complex (GDC) protein were up-regulated (Vincent et al., 2007; Zhang et al., 2010). It has been verified that irrigated and water-stressed plants often show similar values for V_cmax_ and J_max_ (de Souza et al., 2005), and V_cmax_ remained almost unchanged both under water stress and recovery (Galle et al., 2009). In the present study, these two effects were observed. Firstly, the reduction of A_N_, *g*_s_, and *g*_m_ were accompanied by reductions in V_cmax_ and J_max_ under water stress conditions (Table 1), but the magnitudes of the reduction of V_cmax_ and J_max_ were much smaller than for A_N_, *g*_s_, and *g*_m_. Secondly, A_N_, *g*_s_, and *g*_m_ remained below the levels of the control plants after 3 days of re-watering, but V_cmax_ and J_max_ were totally restored and even surpassed the control value. In accordance with the reports by Tang et al. (2013) and Galle et al. (2011), the increased V_cmax_ in response to the decreased C_c_ and *g*_m_ improved the photosynthetic capacity, and contributed to the maintenance of photosynthesis under water stress treatment, most notably, during recovery.

Indeed, chlorophyll fluorescence analysis also supported this conclusion. After 4 days of water stress treatment, decreases in J_flu_ and ΦPSII were observed, and indicated a decline in the quantum yield of the electron transport in PSII (de Souza et al., 2013), and limited the synthesis of ATP and the regeneration of RuBP (Lin et al., 2009), which caused the low activation of Rubisco (V_cmax_). However, F_v_/F_m_ was maintained between 0.82 and 0.85 throughout the experiment, indicating that PSII was quite resistant to water stress treatment. In addition, qP, J_flu_, and ΦPSII were fully recovered after 3 days of re-watering. The above results suggest that water stress inevitably damaged the light reactions, with possible damage to PSII functionality, but the occurrence of damage did not seem to be irreversible. The same trend has already been shown in some species, particularly in those showing increased paraheliotropism in response to water stress (Pastenes et al., 2005; Wang et al., 2012).

### Photosynthetic limitations during water stress and recovery

The photosynthetic limitation analysis showed that S_L_ was negligible, and that MC_L_ and B_L_ were the main limitation factors after 4 days of water stress treatment (Table 2). This indicates that 4 days of water stress treatment did not affect the opening of the stomata, the internal transfer of CO_2_, or the photosynthetic rate. With the continuing water stress treatment, A_N_, *g*_s_ and *g*_m_ declined rapidly and significantly by day 8. At the same time, S_L_ and MC_L_ increased markedly, and the increase of MC_L_ was far greater than that of S_L_, and thus MC_L_ became the main important limitation factor under water stress conditions. It is worth noting that *g*_m_ of *R. delavayi* was remarkably smaller than *g*_s_, even in the control plants, although the differences became smaller as the water stress intensified. This phenomenon has been widely described in woody plants, especially for sclerophyllous species (Hanba et al., 2002; Warren and Dreyer, 2006; Galmés et al., 2007), although not in all cases. Previous studies indicate that leaf anatomical characteristics have an important role in driving the photosynthesis and the potential *g*_m_ (Tosens et al., 2012; Tomás et al., 2013). In particular, CO_2_ diffusion through the mesophyll tissues is significantly limited by the cell wall thickness and the chloroplast envelope. Besides, the chloroplast surface area exposed to intercellular air spaces per leaf area have been proposed as major determinants of differences in *g*_m_ between species (Terashima et al., 2011; Tomás et al., 2013). Our previous study found that leaf anatomical characteristics of *R. delavayi* may have effects on *g*_m_ and CO_2_ transfer, including traits such as smaller stomata, higher stomatal density, and a higher ratio of palisade to spongy mesophyll tissues (Cai et al., 2014). Also, other leaf anatomical characteristics might have contributed to the regulation of *g*_m_, emphasizing the need for further investigations.

After re-watering for 1 day, *g*_m_ was almost complete recovery. *g*_s_ showed some recovery and not fully restored to the control level after 3 days of re-watering. The recovery extent largely depended on the species, from almost null (e.g., *Pistacia lentiscus*) to almost complete (e.g., *Limonium magallufianum*) after re-watering for 1 day (Galmés et al., 2007). The limitation analysis performed for recovery data revealed that the recovery of photosynthesis was most affected by MC_L_ rather than by S_L_ and B_L_ (Table 2). This result contrasts with the results of Ennahli and Earl (2005), who showed in cotton that, after severe water stress, recovery 24 h after re-watering was mostly caused by biochemical limitations, while stomatal and mesophyll limitations were almost totally absent. However, our results are in agreement with the results of Galmés et al. (2007), who showed that *g*_m_ was the most important factor limiting photosynthesis recovery after severe water stress treatment, regardless of the plant growth form and leaf anatomy. To our knowledge, there are few reports showing that limited recovery of *g*_m_ is the most important factor limiting photosynthesis recovery after a severe water stress. Our findings strongly reinforced the important role of *g*_m_ in response of photosynthesis in *R. delavayi* plants under water stress and recovery, and indicate the necessity of better understanding the regulation of *g*_m_, which likely depend on the metabolism related to environmental conditions and leaf anatomy.

In conclusion, 4 days of water stress had little effect on A_N_, *g*_s_, and *g*_m_ of *R. delavayi*. After 8 days of water stress treatment, a marked stomatal closure and a decrease in A_N_ and *g*_m_ were observed. After re-watering, *g*_m_ recovered faster than A_N_ and *g*_s_ did. Photosynthetic limitation revealed that down-regulation of *g*_m_ was the main limitation factor both under water stress and recovery.

### Conflict of interest statement

The authors declare that the research was conducted in the absence of any commercial or financial relationships that could be construed as a potential conflict of interest.

## Supplementary material

Table S1 **Leaf relative water content (RWC) of ***R. delavayi*****.

Figure S1 **The leaf adaxial surface of ***R. delavayi*****.

Figure S2 **The leaf abaxial surface of ***R. delavayi*****.

Figure S3 **Recovery of the ***R. delavayi*** during the experiment was repeated in 2015, the red circles indicate survival plants after re-watering**. Re-watering was done when the stomatal conductance reached 0.02 mol CO_2_ m^−2^s^−1^ and the drought continued for 4 days.
