# Supplementary material for: Photosynthetic Response of an Alpine Plant, Rhododendron delavayi Franch, to Water Stress and Recovery: The Role of Mesophyll Conductance
Source: Front Plant Sci. 2015 Dec 8;6:1089. doi: 10.3389/fpls.2015.01089 (PMC4672053; doi:10.3389/fpls.2015.01089)
Supplement: Table S1 — Leaf relative water content (RWC) of R. delavayi. [file Table1.PDF]

Table S1. Leaf relative water content (RWC) of *R. delavayi*

No	FW（g）	TW（g）	DW (g)	RWC (%)
1	4.10	4.72	1.70	79.47
2	4.15	4.98	1.66	75.00
3	5.26	5.90	1.95	83.80
4	4.14	5.03	1.38	75.62
5	4.74	5.74	1.48	76.53
6	4.24	4.82	1.40	83.04
7	5.03	5.44	1.73	88.95
8	3.90	4.60	1.66	76.19
9	3.57	4.21	1.47	76.64
10	3.79	4.36	1.17	82.13
Mean				79.74
