# Supplementary figures and images for: Photosynthetic Response of an Alpine Plant, Rhododendron delavayi Franch, to Water Stress and Recovery: The Role of Mesophyll Conductance
Source: Front Plant Sci. 2015 Dec 8;6:1089. doi: 10.3389/fpls.2015.01089 (PMC4672053; doi:10.3389/fpls.2015.01089)

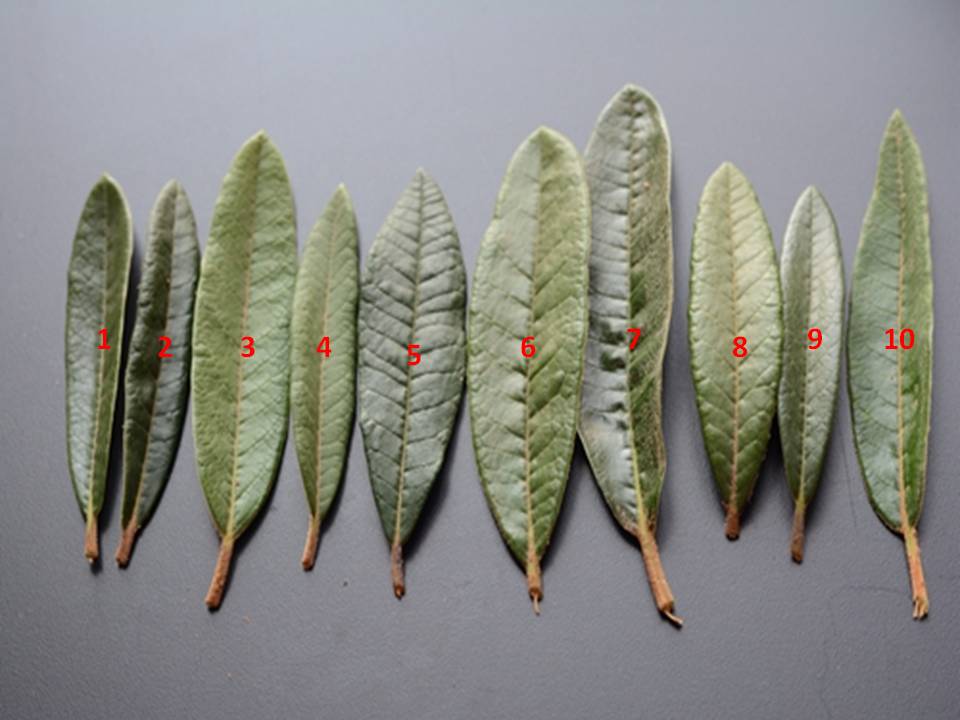

Supplement: Figure S1 — The leaf adaxial surface of R. delavayi. [file Image1.JPEG]

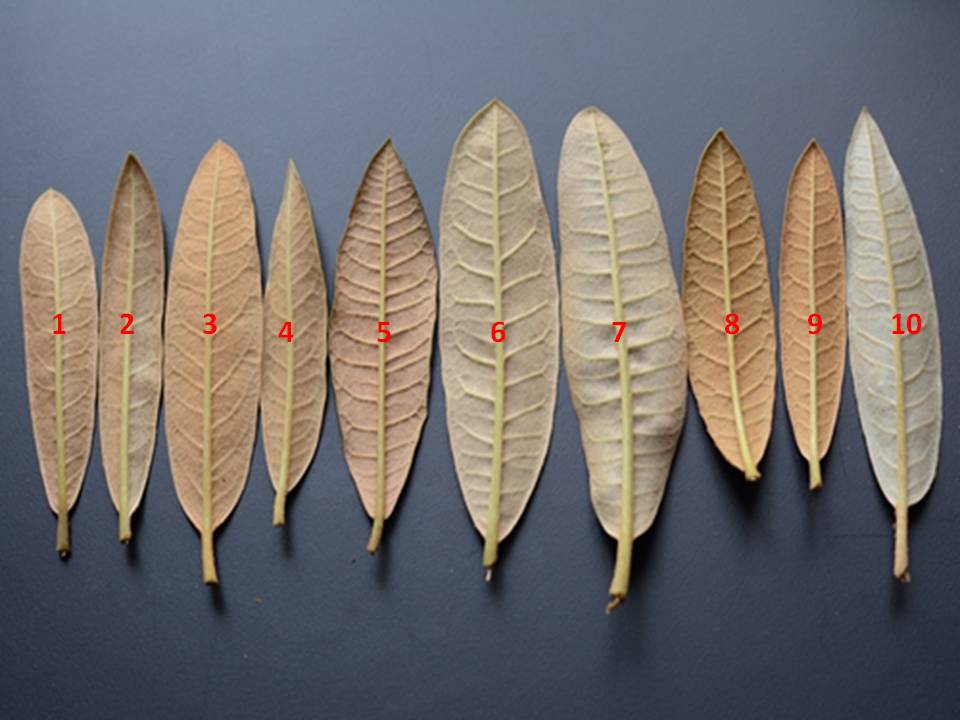

Supplement: Figure S2 — The leaf abaxial surface of R. delavayi. [file Image2.JPEG]

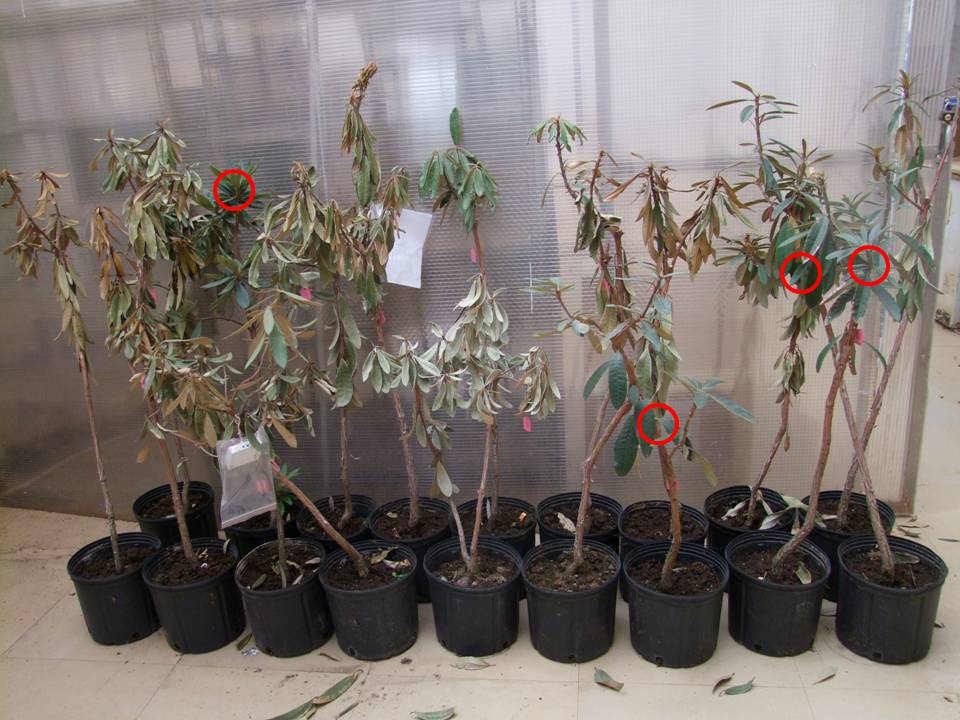

Supplement: Figure S3 — Recovery of the R. delavayi during the experiment was repeated in 2015, the red circles indicate survival plants after re-watering. Re-watering was done when the stomatal conductance reached 0.02 mol CO2 m−2s−1 and the drought continued for 4 days. [file Image3.JPEG]
